# The double-edged role of creativity: silence mechanisms linking workplace incivility to counterproductive work behavior

**DOI:** 10.3389/fpsyg.2026.1788243

**Published:** 2026-03-12

**Authors:** Ailin Qiu, Yuming Liu, Zhaoqi Li, MyeongCheol Choi

**Affiliations:** 1 Department of Economics and Management, Neijiang Normal University, Neijiang, China; 2 Department of Business Management, Gachon University, Seongnam, Republic of Korea; 3 Department of Information Engineering, Shandong Vocational College of Light Industry, Zibo, China

**Keywords:** workplace incivility, creativity, acquiescent silence, defensive silence, counterproductive work behavior, conservation of resources theory

## Abstract

Drawing on conservation of resources theory, this study examines how workplace incivility influences CWB through employee silence in technology-mediated work environments while considering the moderating role of employee creativity. Using survey data collected from 487 Chinese employees working in digitally integrated enterprises, this study develops and tests a moderated mediation model in which acquiescent silence and defensive silence function as two parallel mediating mechanisms linking workplace incivility and CWB. The results indicate that workplace incivility significantly increases CWB through both forms of silence, suggesting that employee silence represents a key behavioral pathway through which incivility contributes to individual psychological resource depletion. Moreover, creativity exhibits differential moderating effects across the two indirect pathways. Specifically, creativity strengthens the indirect effect of workplace incivility on CWB via acquiescent silence while weakening the indirect effect via defensive silence. These findings suggest that the behavioral consequences of creativity depend on the type of silence employees adopt in response to incivility. By distinguishing between acquiescent silence and defensive silence, this study advances understanding of employee behavioral response mechanisms in technology-mediated work environments and highlights the context-dependent role of creativity as a psychological resource.

## Introduction

In high pressure environments driven by efficiency and innovation, particularly within rapidly evolving digital ecosystems, modern organizations often overlook a subtle but pervasive form of “soft violence”: workplace incivility. Interactions among employees have significantly increased with the widespread adoption of digital communication tools, such as instant messaging applications, enterprise collaboration platforms, and AI-enabled office systems. However, blurred worklife boundaries, reduced face-to-face communication, and algorithmic performance pressures have contributed to a growing trend of incivility that is increasingly hidden, digitalized, and structurally embedded. Such behaviors often manifest as rudeness, thoughtlessness, ambiguity, arrogance, and disrespect (Lim and Teo, 2009), leaving employees feeling ignored, powerless, and insignificant (Giumetti et al., 2012). Although prior research has primarily focused on workplace incivility in traditional face-to-face settings, its expressions within technology-mediated interaction contexts have become increasingly diverse. Such behaviors may include delayed or ignored digital responses (e.g., “read-but-not-reply”), online shaming, perceived unfairness in task coordination, and platform-based monitoring practices (Ju and Pak, 2025; Torres et al., 2024). Compared with conventional interaction environments, these technology-mediated expressions of incivility are often characterized by greater subtlety and interpretive ambiguity (Lim and Teo, 2009).

Accumulating evidence suggests that work environments characterized by high reliance on digital communication and technological tools are frequently accompanied by elevated psychological strain and technology-related stress. Reports such as the Microsoft Work Trend Index indicate that many employees experience time pressure and digital overload, reflecting the stress associated with persistent connectivity and communication demands. Empirical studies further show that incivility occurs at relatively high rates in computer-mediated communication (Lim and Teo, 2009), with employees frequently encountering rude or disrespectful online interactions (Giumetti et al., 2012). In addition, technostress has emerged as a critical stressor in modern work environments (Li et al., 2025). Cross-national studies demonstrate that technostress significantly increases psychological tension, emotional exhaustion, and dysfunctional behavioral responses (Tarafdar et al., 2019). Technology-induced stress experiences may further intensify emotional exhaustion, thereby increasing the likelihood of counterproductive work behavior (CWB) (Fuglseth and Sørebø, 2014). Within such interaction contexts, workplace incivility may function as a persistent interpersonal stressor that exacerbates employees’ resource depletion processes.

Moreover, CWB represents another serious concern that poses significant threats to organizational performance and sustainability. CWB refers to actions that may harm organizations or their members, including abuse, production deviance, sabotage, withdrawal, and theft (Fan et al., 2023). In contemporary work settings, CWB manifests in diverse forms, such as information hiding, data manipulation or destruction, malicious system operations, and misuse of digital resources (Reizer et al., 2020). Prior research indicates that CWB is closely associated with individual traits (e.g., narcissism or low self-control), technostress, remote collaboration conflicts, and perceptions of organizational injustice (Salgado et al., 2013). As digital monitoring and algorithmic performance evaluation intensify, employees’ emotional burdens increase substantially, further elevating the risk of CWB (Spector and Fox, 2002; Fox et al., 2001). Therefore, in technology-mediated work environments, building high-quality work experiences, reducing incivility, and establishing psychologically supportive climates are crucial for preventing CWB (Carpenter et al., 2021).

Workplace incivility frequently leads to employee silence. In digital environments, silence may be even more pronounced when employees hesitate to express genuine concerns due to fear, marginalization, algorithmic monitoring, and emotional distress from digital communication (Benbow et al., 2024; Kougiannou and Holland, 2022). Silence is a collective phenomenon that inhibits the detection of organizational issues and impedes pathways to innovation (Morrison and Milliken, 2000). According to Dyne et al., (2003), silence includes acquiescent and defensive silence, which differ substantially in the context of digital work.

Acquiescent silence stems from powerlessness and learned helplessness. In digital systems, when employees repeatedly offer suggestions that are unacknowledged or rejected by algorithms, they gradually stop speaking up and ultimately enter a passive compliance state (Andrieu et al., 2024). Such silence is particularly problematic in highly digitalized organizations because it undermines employees’ psychological well-being and organizational identification (Weiss and Zacher, 2025), suppresses digital innovation, and reduces creativity and proactive behavior (Jahanzeb and Raja, 2024). However, high power distance orientation, destructive leadership, technostress overload, and chronic digital fatigue reinforce acquiescent silence (Lam and Xu, 2019; Jungst and Verbeeck, 2025).

In contrast, defensive silence arises from fear and strategic avoidance. Moreover, with continuous digital monitoring, task transparency, and real-time performance visibility, employees may become cautious and fear that voicing concerns may lead to negative, traceable consequences. This form of silence is common in highly digitalized, politically intense, and AI-driven management environments characterized by opacity and uncertainty (Kiewitz et al., 2016; Lam and Xu, 2019). Although defensive silence may help employees avoid short-term interpersonal or political risks, it damages psychological contracts, erodes trust, and leads to emotional exhaustion, interpersonal deviance, and reduced job satisfaction (Jahanzeb et al., 2018; Jahanzeb and Fatima, 2018). In industries highly dependent on digital collaboration and service innovation, defensive silence further suppresses creativity and undermines sustainable competitiveness in digital ecosystems (Zou et al., 2025).

However, silence due to incivility does not always lead to CWB. Creativity may function as a crucial moderating factor in this process. As a comprehensive psychological resource, creativity reflects cognitive flexibility and problem-solving capabilities, helps employees mitigate digital stress, restores a sense of meaning, and maintains work satisfaction (George, 2007; Kim et al., 2009). Nevertheless, the effects of creativity differ across silence types: employees exhibiting acquiescent silence lack the motivation to act, making it difficult for creativity to translate into behavior (Maqbool et al., 2019), whereas employees exhibiting defensive silence retain the intention to participate. For them, creativity provides alternative strategies, strengthens digital resilience and perceived efficacy, helps buffer negative emotions, and resists hostile CWB (Xu et al., 2022). In digital ecosystems, creativity is a psychological resource and fundamental driver of sustainable organizational innovation, making its buffering effect particularly significant.

Although prior research has examined the relationships among workplace incivility, employee silence, creativity, and CWB, several important gaps remain. First, many studies overlook the motivational heterogeneity of employee silence, frequently combining different silence forms and thereby obscuring their distinct psychological mechanisms. Second, although creativity has been widely regarded as an important antecedent of positive workplace behaviors, it has rarely been interpreted from a resource regulation and restoration perspective. Third, these variables have not yet been systematically integrated within a coherent theoretical framework under technology-mediated interaction contexts. As technology-mediated work patterns become increasingly prevalent, there is a pressing need to re-examine employee behavioral mechanisms from a “resource depletion–protection–recovery” perspective.

To address these gaps, this study draws upon conservation of resources (COR) theory to develop a parallel mediation model, examining how workplace incivility influences CWB via acquiescent silence and defensive silence, while incorporating creativity as a moderating variable. The empirical analysis focuses on employees working in industries characterized by high reliance on digital communication and technology-mediated coordination in China. This study not only advances our understanding of the differentiated mechanisms linking workplace incivility and employee silence but also offers important implications for managerial practices in technology-mediated work environments.

## Theory and hypotheses

### Conservation of resources theory

COR theory posits that individuals are primarily motivated to avoid resource loss and tend to prioritize resource-protection strategies under conditions of sustained stress, rather than actively pursuing resource gains (Halbesleben et al., 2014). When employees perceive their resources to be scarce or threatened, their behavioral orientation often shifts from active engagement to risk avoidance and loss minimization in order to prevent further depletion of psychological and social resources (Zhang et al., 2025). In digitalized work contexts, this resource-protection orientation becomes particularly salient. Unlike traditional face-to-face interactions, digital work incivility often manifests in depersonalized, continuously visible, and difficult-to-contest forms, such as algorithmic task allocation, platform-based monitoring, and communication characterized by delayed or absent feedback. These behaviors are not isolated incidents but are structurally embedded in work systems, rendering digital incivility a chronic and cumulative threat to employees’ resources (Halbesleben et al., 2014; Farkash et al., 2022).

According to COR theory, when resource threats persist over time and recovery channels are limited, employees are more likely to adopt defensive, loss-minimization responses rather than constructive investment behaviors (Li and Song, 2024; Niessen and Jimmieson, 2016). In the context of digital work incivility, such defensive orientations are reflected in reduced voice, avoidance of interaction, and lower levels of participation—that is, employee silence. As resource depletion intensifies and opportunities for recovery diminish, some employees may further engage in CWB, such as withdrawal or information withholding, as a means of compensating for or rebalancing threatened resources (Hobfoll et al., 2018; Li and Jo, 2025). Thus, COR theory provides a clear and integrative framework for understanding how digital work incivility translates into counterproductive work behavior through silence as a defensive mechanism.

### Workplace incivility and counterproductive work behavior

Workplace incivility refers to low-intensity deviant behavior characterized by rudeness, disrespect, and violations of norms for mutual respect, with ambiguous intent to harm (Cortina et al., 2001). In digitalized workplaces, such incivility extends beyond traditional face-to-face interactions and frequently manifests through technology-mediated exchanges. These behaviors include delayed or ignored online responses, exclusion from digital communication channels, public criticism in virtual spaces, opaque algorithmic task assignments, and platform-based monitoring practices shaped by biased decision rules (Kadolkar et al., 2025; Pothuganti, 2024).

Importantly, workplace incivility functions as a chronic interpersonal stressor that threatens employees’ socio-emotional resources, psychological safety, and perceived respect (Vasconcelos, 2023). Compared with traditional workplace settings, digital environments may intensify the experience of incivility due to reduced interpersonal cues, increased visibility, and heightened communication traceability. As a result, employees exposed to workplace incivility are more likely to engage in CWB.

Prior research provides substantial empirical support for this relationship. For instance, coworker incivility triggers negative emotional reactions and reduces work engagement, which may lead employees to withdraw from digital collaboration or withhold information, thereby increasing CWB (Sakurai and Jex, 2012). Similarly, supervisor incivility, whether occurring offline or through digital communication, is positively associated with employees’ CWB. However, this negative impact may be partially mitigated when employees possess strong personal resources, such as internal locus of control or adaptive digital job crafting strategies (Naeem et al., 2024).

Moreover, contextual conditions may amplify the behavioral consequences of incivility. When incivility levels vary substantially within teams and members generally remain silent—particularly in digital contexts where uncivil behaviors are more visible yet less openly discussed—individual experiences of incivility may escalate into destructive behavioral responses (Mao et al., 2019; Qiu et al., 2025). Task structures also matter. Under conditions of high digital task interdependence, employees experience stronger strain reactions, and female employees may be particularly vulnerable to the stress induced by incivility, further increasing the likelihood of CWB (Welbourne and Sariol, 2017).

From the perspective of COR theory (Hobfoll, 1989), workplace incivility represents a persistent resource-draining condition that threatens employees’ cognitive, emotional, and social resources. Employees encountering repeated disrespect, digital neglect, and unfair treatment experience sustained socio-emotional resource depletion, a process further intensified by digitally mediated conditions characterized by constant connectivity, platform surveillance, and communication overload. As resources diminish, individuals develop self-protective motivations, which may trigger defensive regulatory responses such as silence, avoidance, and reduced cooperation, or more maladaptive reactions such as resistance and counterproductive behavior (Hobfoll et al., 2018).

Under conditions of perceived resource scarcity, CWB—including intentional work delays, digital withdrawal, improper use of digital tools, and withholding key information—may function as a maladaptive resource regulation strategy aimed at offsetting perceived losses of control, dignity, or fairness. Employees may seek to restore psychological balance or regain a sense of control by disengaging from work or undermining organizational interests.

Accordingly, this study proposes the following hypothesis:

*H1*: Workplace incivility is positively related to CWB.

### The mediating role of silence

Workplace incivility is a major antecedent of employee silence and indirectly contributes to CWB through distinct silent pathways. In digitalized work environments, this process becomes more complex. The immediacy, traceability, and visibility of digital communication heighten employees’ perceived risk of expressing themselves and intensify the psychological strain associated with uncivil interactions. Based on their underlying motivational drivers and psychological mechanisms, silence can be categorized into two types: acquiescent and defensive silence (Dyne et al., 2003).

First, acquiescent silence arises from “learned helplessness” of employees after repeated experiences of being ignored or devalued (Knoll et al., 2019). In digital settings, such disregard often takes the form of being excluded from communication channels, being left on “read,” and receiving delayed or absent online responses. When employees encounter managerial incivility, such as sarcasm in group chats, dismissive comments in virtual meetings, and public criticism in internal platforms, they perceive their voices as undervalued or systematically suppressed. This perception reduces the motivation and confidence to speak out.

This pattern aligns with COR theory (Hobfoll, 1989). Attempting to express themselves repeatedly results in cognitive, emotional, and social resource depletion, exacerbated by the heightened visibility and potential misinterpretation inherent in digital communication, and employees withdraw from voice behavior to conserve the remaining resources. Such withdrawal manifests as acquiescent silence. This silence further weakens organizational identification, deteriorates digital collaboration climates, and impairs organizational problem detection and innovation capacity (Ballekura et al., 2025; He et al., 2019), ultimately increasing the likelihood of CWB (Gupta et al., 2025).

Second, defensive silence reflects a strategic withholding of expression in which employees remain silent out of fear of retaliation, conflict, or negative evaluation (Srivastava et al., 2023). Digital environments amplify these fears, as online messages are recordable, transferable, and permanently traceable, and digital monitoring systems and platform-based performance logs intensify the sense of being constantly observed (Grisold et al., 2024). Supervisors’ digital incivility, such as online humiliation or harsh criticism during virtual meetings, heightens employees’ perceptions of depersonalization and encourages silence as a means of self-protection (Abid et al., 2025). Defensive silence is particularly prevalent in environments characterized by asymmetric digital power dynamics and strong organizational politics (Kiewitz et al., 2016; Mohammed Soliman et al., 2024). Especially, women and minority employees, who often experience higher expression risks and greater online visibility, are likely to adopt defensive silence (Benbow et al., 2024; Bain et al., 2025).

Moreover, both forms of silence, whether motivated by passive resignation or strategic self-protection, are closely associated with CWB. Employees who exhibit acquiescent silence tend to withdraw, disengage, and reduce their cooperation. In contrast, those engaged in defensive silence may develop emotional exhaustion or engage in knowledge hiding, thereby increasing the potential for retaliatory or avoidant forms of CWB (Qi and Ramayah, 2022). Consequently, workplace incivility indirectly heightens employees’ propensity for CWB through these two psychological pathways. Based on the above analysis, this study proposes the following hypotheses:

*H2*: Acquiescent silence mediates the relationship between workplace incivility and CWB.

*H3*: Defensive silence mediates the relationship between workplace incivility and CWB.

### The moderating role of creativity

In recent years, creativity has been recognized as a critical psychological resource that drives organizational development and has been widely applied to explain the mechanisms of employee behavioral regulation (Shi et al., 2020; Xu et al., 2022). In general, employees with higher creativity tend to exhibit fewer forms of CWB, such as sabotage, theft, and work withdrawal (Harari et al., 2018). Meta-analytic evidence also confirms a significant negative correlation between creativity and CWB: Employees with stronger creative capabilities are more likely to contribute constructively rather than engage in behaviors that undermine organizational interests (Harari et al., 2018).

However, creativity does not consistently function as a positive resource in highly digitalized work environments. Digital platforms often generate communication over-load, opaque algorithmic task assignments, constant online monitoring, and increased information complexity within virtual collaborations, which amplify employees’ re-source depletion and psychological strain. Under these conditions, creativity may exhibit a “double-edged sword” effect.

Along the pathway of acquiescent silence, employees repeatedly experience ineffective expression, neglected opinions, and algorithmic rejection in digital systems, leading to a state of learned helplessness and the continuous depletion of voice opportunities, a sense of control, and social resources (Dyne et al., 2003). According to the COR theory (Hobfoll, 1989), such silence represents a state of resource exhaustion. Employees with higher creativity, owing to their heightened cognitive sensitivity and stronger need for expression, are more likely to perceive digital systems as unfair, opaque, and communication blocking, thereby experiencing stronger cognitive–emotional conflict. However, when this strain cannot be relieved through formal channels, creativity may instead facilitate strategic forms of CWB, such as delaying digital tasks, reducing online responsiveness, withholding information, and resisting digital workflows, as a means of restoring psychological balance and regaining a sense of control (Ng and Yam, 2019; Hameed et al., 2025).

Conversely, in the defensive silence pathway, employees remain silent not because they have abandoned expression, but because they fear conflict, retaliation, or leaving digital traces under platform monitoring (Qi and Ramayah, 2022; Wynen et al., 2020). In this high-risk digital context, employees with higher creativity typically possess greater abilities in situational reframing, alternative strategy generation, and emotional regulation. These cognitive advantages enable them to navigate digital constraints through informal communication, asynchronous coordination, or virtual tasks reorganization, thereby reducing psychological pressure and mitigating deviant behavior (Santiago-Torner, 2024; Santiago-Torner et al., 2025).

Therefore, in digitalized work environments, creativity exhibits an apparent “dual moderating effect” across different silence mechanisms: it amplifies stress and deviant tendencies within resource-depleting acquiescent silence. Still, it serves as a buffering and protective factor within resource-conserving defensive silence. Based on this logic, this study proposes that creativity will function as either a risk amplifier or protective attenuator across different silence pathways.

*H4*: Creativity positively moderates the relationship between acquiescent silence and counterproductive work behavior, such that the positive effect of acquiescent silence on CWB is stronger among highly creative employees.

*H5*: Creativity negatively moderates the relationship between defensive silence and counterproductive work behavior, such that the positive effect of defensive silence on CWB is weaker among highly creative employees.

### Moderated mediation models

Based on Hypotheses 1–5, we integrated the moderated mediation framework to formulate and examine our theoretical model. Our model posits that acquiescent and defensive silences serve as dual mediating mechanisms capable of translating interaction effects involving creativity into CWB. Accordingly, we propose that the strength of indirect pathways linking workplace incivility to CWB via acquiescent and defensive silence is contingent on employee creativity. For employees with high levels of creativity, exposure to workplace incivility is expected to lead to greater acquiescent silence, which, in turn, results in increased levels of CWB. For employees with high creativity, exposure to workplace incivility is expected to lead to greater defensive silence, thereby reducing CWB. Therefore, this study proposes the following conditional indirect effect hypotheses:

*H6*: Exposing employees with high creativity to workplace incivility leads to greater acquiescent silence, resulting in higher levels of CWB.

*H7*: Exposing employees with high creativity to workplace incivility leads to greater defensive silence, resulting in reduced CWB.

**Figure 1:**
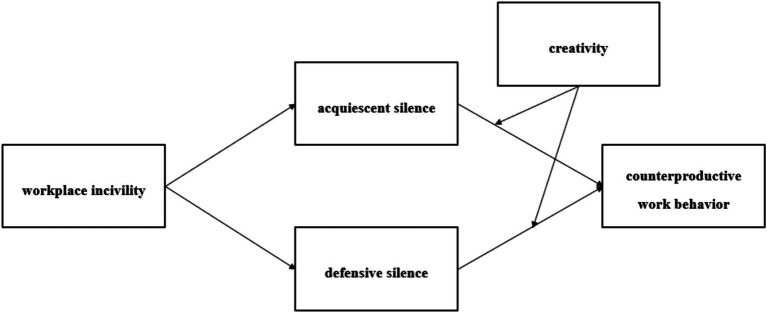
Research model.

The hypotheses presented above form the basis of our conceptual model (Figure 1).

## Methods

### Sample and data collection

This study employed an online questionnaire to collect data from employees working in digitally integrated workplaces across Guangdong, Sichuan, and Shandong provinces in China. Data collection was conducted over a two-month period. The research sample was primarily drawn from industries that heavily rely on digital communication tools and technology-mediated coordination processes, including information technology services, digital manufacturing, platform-based service firms, and organizations widely adopting mobile or AI-assisted work systems.

This study adhered to the ethical principles outlined in the Declaration of Helsinki. Ethical review and approval were waived in accordance with Article 34 of the Gachon University Institutional Review Board (IRB) Standard Operating Guidelines, as the study employed an anonymous questionnaire design involving minimal risk, voluntary participation, and no collection of personally identifiable information. Prior to data collection, respondents were fully informed about the purpose of the study, the survey procedures, confidentiality safeguards, and their rights as research participants. Informed consent was obtained before participation. Participation was entirely voluntary, and respondents were free to discontinue at any time. All data were collected anonymously and analyzed in aggregate form to ensure participant confidentiality.

Participation followed a voluntary basis, and no monetary or material incentives were provided. To minimize evaluation apprehension and reduce potential social desirability bias, several procedural remedies were implemented during the questionnaire design and administration stages. Specifically, (1) survey items avoided emotionally charged or evaluative language, (2) the names of the focal constructs were masked to reduce respondents’ cognitive inference and consistency tendencies, and (3) all items were presented using neutral and descriptive wording.

A total of 600 questionnaires were distributed, and 521 responses were initially obtained. Following established survey research practices, a systematic data screening procedure was conducted. Questionnaires were excluded if they exhibited (a) missing values on key variables, (b) logically inconsistent or mismatched responses, or (c) extreme response patterns. Extreme responses were identified using two criteria. First, responses displaying uniform answering patterns across scale items (e.g., identical ratings for nearly all items) were removed to eliminate mechanically completed questionnaires or inattentive responses. Second, responses with completion times substantially shorter than the minimum reasonable threshold were excluded to address unusually rapid answering behavior. After applying these screening and quality control procedures, 487 valid responses were retained, yielding an effective response rate of 80.7%.

Among the 487 valid respondents, 244 were male (50.1%), and 243 were female (49.9%). In terms of age distribution, 106 respondents were 25 years and under (21.77%), 194 were aged between 26 and 30 (39.84%), 108 were aged between 31 and 35 (22.18%), 59 were aged between 36 and 40 (12.11%), 13 were aged between 40 and 50 (2.67%), and 7 were aged 50 years or above (1.44%). The majority of the respondents held junior college or undergraduate degrees, totaling 391 individuals (80.29%). In terms of tenure, more than half of the respondents had fewer than 5 years of work experience (55.85%), while 215 respondents (44.15%) had more than 5 years of work experience.

### Measures

The measurement scales used in this study were derived from previously validated instruments. A double-translation process was employed to ensure linguistic and conceptual accuracy.

Workplace incivility was measured using a modified version of the Workplace Incivility Scale (Cortina et al., 2001). The scale was adapted to capture employees’ exposure to uncivil behaviors in digital communication environments. Consistent with prior research, participants were asked to indicate how frequently they experienced uncivil behaviors during the past month. Responses were recorded on a 5-point frequency scale (1 = never, 2 = rarely, 3 = sometimes, 4 = often, 5 = very often). Sample items include: “Were demeaning or derogatory remarks made about you in online communication environments?” and “Were you addressed in unprofessional terms in online communication environments?”(Cronbach’s *α* = 0.843).

Acquiescent silence was measured using the four-item scale developed by Dyne et al. (2003). Consistent with the self-report design of this study, items were administered in first-person format. Responses were recorded using a 5-point Likert scale (1 = strongly disagree to 5 = strongly agree). Sample items include: “I am unwilling to speak up with suggestions for change because I feel disengaged,” and “I passively withhold ideas due to resignation.” (Cronbach’s α = 0.847).

Defensive silence was measured using the five-item scale developed by Dyne et al. (2003). Items were presented in first-person format. Responses were recorded using a 5-point Likert scale (1 = strongly disagree to 5 = strongly agree). Sample items include: “I do not speak up with suggestions for change because I am afraid,” and “I withhold relevant information due to fear.” (Cronbach’s α = 0.878).

Creativity was assessed using the six-item scale proposed by Madjar et al. (2011). Responses were recorded using a 5-point Likert scale (1 = strongly disagree to 5 = strongly agree). Sample items include: “Uses previously existing ideas or work in an appropriate new way,” and “Easily modifies previously existing work processes to suit current needs.” (Cronbach’s α = 0.858).

Counterproductive work behavior was measured using the four-item scale developed by Yang and Diefendorff (2009). Responses were recorded using a 5-point Likert scale (1 = strongly disagree to 5 = strongly agree). Sample items include: “Took an additional or longer break than allowed,” and “Intentionally worked slower than one could have worked.” (Cronbach’s α = 0.846).

### Statistical analysis

SPSS 28.0 and Amos 26.0 were used for data processing. Harman’s single-factor analysis was used to test the degree of common method bias. Descriptive analyses were conducted to describe demographic variables. Pearson correlations were calculated among workplace incivility, acquiescent silence, defensive silence, creativity, and CWB. This study employed a multiple regression analysis and SPSS PROCESS (with bootstrap resampling set at 5000 iterations) to test the hypotheses. Gender, age, education, and tenure were included as control variables.

## Results

### Common method bias

This study conducted Harman’s one-factor test to assess the possibility of common method bias. After performing a rotated factor analysis on all items in the questionnaire, a significant common method bias was indicated if a single factor emerged or the first factor accounted for most of the variance. The first-factor interpretation rate was only 34.622%, which was lower than the standard requirement of 40%, indicating no serious systematic bias in the questionnaire result.

In addition, following the full collinearity assessment approach, variance inflation factor (VIF) values were examined to further evaluate potential common method variance. The VIF values ranged from 1.115 to 1.759, all of which were well below the recommended cutoff value of 3.3, indicating that common method bias was not a significant concern in this study.

### Reliability and validity tests

As shown in Table 1, the composite reliability (CR) values for all constructs exceeded 0.7, and the average variance extracted (AVE) values were greater than 0.5. Confirmatory factor analysis (CFA) was performed using AMOS 26.0 to further assess discriminant validity, and the results are detailed in Table 2. The model demonstrated excellent fit to the data: *χ*^2^ = 375.284, *χ*^2^/df = 1.706, RMSEA = 0.038, GFI = 0.933, CFI = 0.971, IFI = 0.971, and TLI = 0.967. All fit indices met the recommended thresholds for an acceptable model fit. Consequently, the model demonstrated strong structural validity, and the variables exhibited satisfactory discriminant validity. Therefore, the discriminant validity of the measurement instrument was confirmed.

**Table 1:** Confirmatory factor analysis.

Variables	Items code	Standardized loadings	AVE	CR	Confirmatory factor analyses
Workplace incivility	WI1	0.776	0.573	0.843	*χ*^2^ = 375.284 *χ*^2^ / df = 1.706 RMSEA = 0.038 GFI = 0.933 CFI = 0.971 IFI = 0.971 TLI = 0.967
	WI2	0.754			
	WI3	0.747			
	WI4	0.751			
Acquiescent Silence	AS1	0.758	0.581	0.847	
	AS2	0.754			
	AS3	0.754			
	AS4	0.783			
Defensive Silence	DS1	0.78	0.590	0.878	
	DS2	0.749			
	DS3	0.766			
	DS4	0.743			
	DS5	0.801			
Creativity	RC1	0.744	0.503	0.858	
	RC2	0.652			
	RC3	0.725			
	C1	0.695			
	C2	0.734			
	C3	0.702			
Counterproductive work behavior	CWB1	0.768	0.579	0.846	
	CWB2	0.763			
	CWB3	0.788			
	CWB4	0.722			

**Table 2:** Descriptive statistics and correlations for study variables.

Variables	1	2	3	4	5	6	7	8	9
1. Gender	1								
2. Age	−0.056	1							
3. Edu	−0.036	−0.005	1						
4. Tenure	0.013	0.655**	−0.033	1					
5. WI	−0.100*	0.008	0.034	0.002	1				
6. AS	−0.139**	0.072	0.120**	0.066	0.444**	1			
7. DS	−0.121**	0.065	0.053	0.084	0.536**	0.560**	1		
8. C	0.099*	−0.032	−0.069	−0.076	−0.178**	−0.274**	−0.292**	1	
9. CWB	−0.178**	0.047	0.012	0.014	0.478**	0.522**	0.512**	−0.270**	1
Mean	1.499	2.384	2.536	1.671	2.769	2.812	2.861	3.622	2.829
SD	0.501	1.118	0.818	0.929	1.172	1.143	1.154	0.899	1.146

*p < 0.05, **p < 0.01. WI, workplace incivility; AS, acquiescent silence; DS, defensive silence, C, creativity; and CWB, counterproductive work behavior.

### Analysis of correlation between variables

The correlation analysis results presented in Table 2 indicate the following significant relationships. Workplace incivility demonstrated significant positive correlations with acquiescent silence, defensive silence, and CWB. Employee creativity was significantly negatively correlated with workplace incivility, acquiescent silence, defensive silence, and CWB. Significant correlations were observed between the five variables. The outcomes of the correlation significance tests satisfied the preconditions for conducting regression analysis.

### Hypothesis testing

Regarding the main effects model, workplace incivility demonstrated a significant positive effect on CWB (*β* = 0.454, *p* < 0.001), providing support for H1. Workplace incivility also exhibited significant effects on acquiescent silence (*β* = 0.420, *p* < 0.001) and defensive silence (*β* = 0.520, *p* < 0.001). Furthermore, acquiescent silence (*β* = 0.382, *p* < 0.001) and defensive silence (*β* = 0.349, *p* < 0.001) exhibited significant effects on CWB. Models 7 and 8 indicated that the mediating variables significantly affected the dependent variable (Table 3). Models 2 and 4 demonstrated that the independent variable had significant positive effects on both mediating variables. This pattern of results provides evidence for mediation effects in the proposed model.

**Table 3:** Results of main and direct effects.

Variables	AS				DS		CWB	
	Model 1	Model 2	Model 3	Model 4	Model 5	Model 6	Model 7	Model 8
Gender	−0.305**	−0.207*	0.278**	−0.157	−0.401***	−0.295**	−0.216*	−0.240**
Age	0.033	0.033	0.001	0.002	0.047	0.047	0.034	0.046
Edu	0.163**	0.144*	0.073	0.050	0.008	−0.012	−0.068	−0.030
Tenure	0.062	0.059	0.108	0.105	−0.016	−0.019	−0.042	−0.056
WI		0.420***		0.520***		0.454***	0.294***	0.273***
AS							0.382***	
DS								0.349***
R^2^	0.038	0.222	0.025	0.300	0.033	0.247	0.360	0.333
F	4.770***	27.429***	3.063**	41.250***	4.130***	31.488***	44.929***	39.997***

*p < 0.05, **p < 0.01, ***p < 0.001. WI, workplace incivility; AS, acquiescent silence; DS, defensive silence; C, creativity; and CWB, counterproductive work behavior.

To address the limitations of hierarchical regression analysis, the model’s mediation effects were examined using the PROCESS macro. The results are summarized in Table 4. The data indicate that acquiescent silence mediates the relationship between workplace incivility and CWB, with an indirect effect of 0.128 (95% CI [0.079, 0.182]). Excluding zero from the confidence interval confirmed that the mediation effect is statistically significant, supporting H2. Defensive silence mediated the relationship between workplace incivility and CWB with an indirect effect of 0.118 (95% CI [0.056, 0.183]). Excluding zero from the confidence interval confirmed that the mediation effect was statistically significant, thereby supporting H3.

**Table 4:** Regression results of mediation effects.

Hypothesis	Effect	Boot SE	Boot LLCI	Boot ULCI
Mediation effects: WI → AS→CWB	0.128	0.026	0.079	0.182
Mediation effects: WI → DS → CWB	0.118	0.032	0.056	0.183

WI, workplace incivility; AS, acquiescent silence; and CWB, counterproductive work behavior.

**Figure 2:**
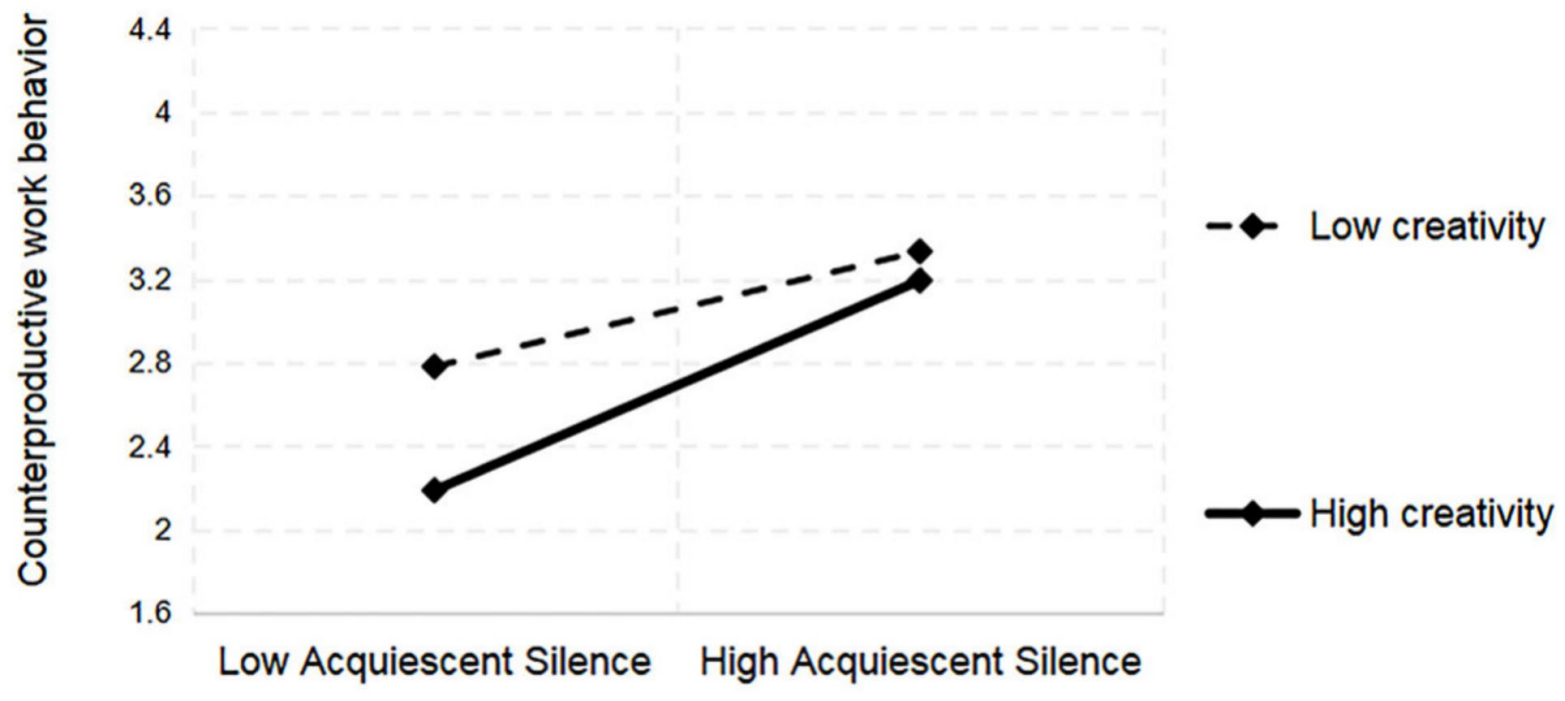
Moderating role of creativity on AS and CWB.

**Figure 3:**
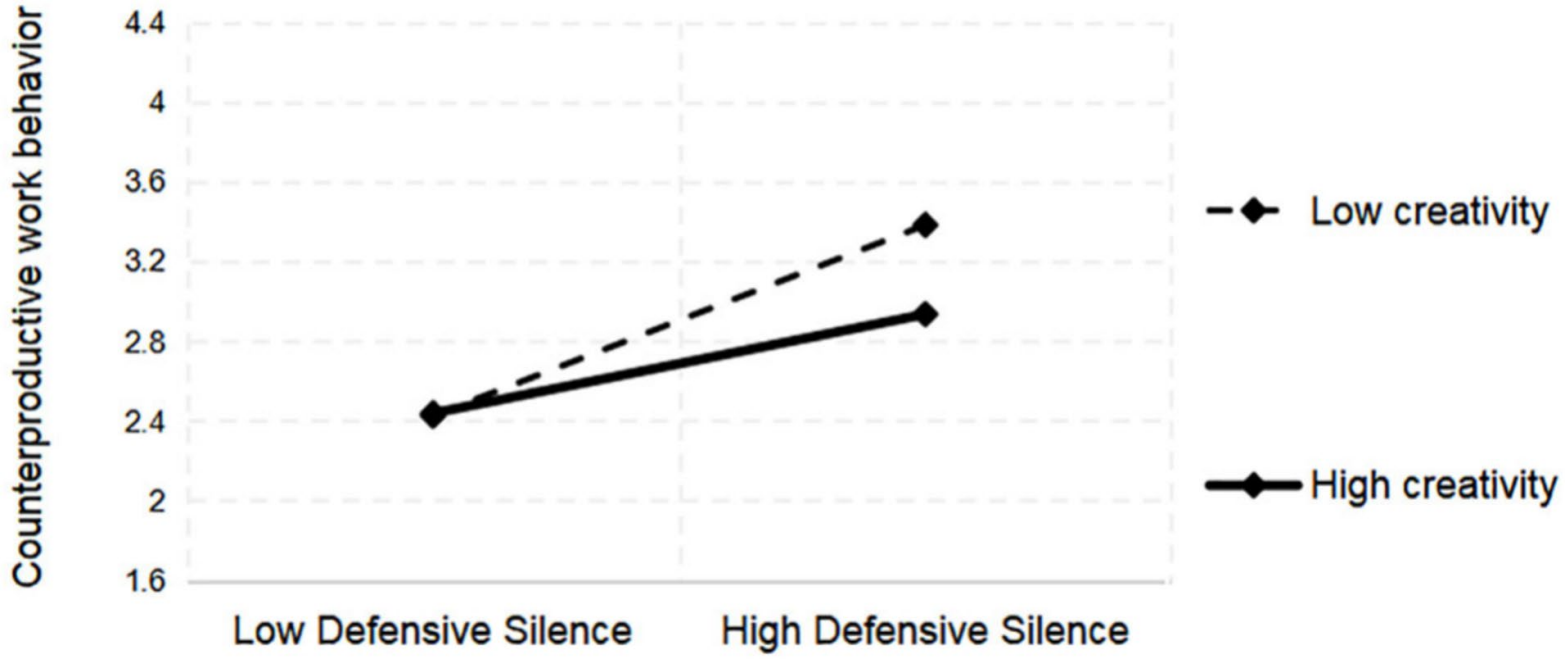
Moderating role of creativity on DS and CWB.

In the moderation effect tests, creativity (C) positively moderated the relationship between acquiescent silence (AS) and CWB. The interaction term “AS × C” had a significant positive effect on CWB (*b* = 0.081, *p* < 0.05). This indicates that, as creativity increased, the positive effect of acquiescent silence on CWB became progressively stronger (Figure 2), supporting H4. Creativity negatively moderated the relationship between DS and CWB. The interaction term “DS × C” had a significant negative effect (*b* = −0.084, *p* < 0.05). This indicates that as creativity increased, the positive effect of defensive silence on CWB progressively weakened (Figure 3), supporting H5. The detailed calculation results are presented in Table 5.

**Table 5:** Regression results of moderating effects.

Hypothesis	Effect	Boot SE	Boot LLCI	Boot ULCI
Moderating effect: AS×C → CWB	0.081	0.039	0.034	0.158
Moderating effect: DS × C → CWB	−0.084	0.040	−0.161	−0.006

AS, acquiescent silence; DS, defensive silence; C, creativity.

Additional analyses were conducted to examine differences in the magnitude of the mediation effects across varying levels of creativity. The results demonstrated that under low creativity conditions, the indirect effect was 0.118 for the “WI → AS → CWB” pathway and 0.219 for the “WI → DS → CWB” pathway. Conversely, under high creativity conditions, the indirect effect increased to 0.190 for the “WI → AS → CWB” pathway and decreased to 0.114 for the “WI → DS → CWB” pathway. Subsequent statistical tests confirmed a significant difference in the mediation effects between high and low creativity levels, indicating a significant moderated mediation. These findings support H6 and H7; the detailed results are presented in Table 6.

**Table 6:** Results of moderated mediation effects.

Hypothesis	Effect	SE	95% Bias-corrected CI	
			Lower	Upper
Test of moderated mediation effects (WI → AS→CWB)				
Index of moderated mediation	0.040	0.019	0.003	0.078
Low C (Mean-1SD)	0.118	0.033	0.058	0.187
High C (Mean+1SD)	0.190	0.031	0.135	0.253
Test of moderated mediation effects (WI → DS → CWB)				
Index of moderated mediation	−0.059	0.021	−0.098	−0.018
Low C (Mean-1SD)	0.219	0.038	0.147	0.296
High C (Mean+1SD)	0.114	0.036	0.048	0.187

WI, workplace incivility; AS, acquiescent silence; DS, defensive silence; C, creativity; CWB, counterproductive work behavior.

## Conclusion

Based on COR theory, this study reveals that workplace incivility in digital ecosystems, often manifested through unread messages, delayed responses, online exclusion, algorithmic unfairness, and platform-based surveillance, significantly accelerates employees’ emotional and cognitive resource depletion, thereby increasing their likelihood of engaging in CWB. The findings emphasize that digitalized incivility is not merely an interpersonal issue, but a systemic threat intensified by high-speed communication, persistent connectivity, and algorithm-driven work environments.

First, workplace incivility significantly and positively predicts CWB. Under digital working conditions, where communication is accelerated and monitoring is pervasive, employees experience intensified psychological vulnerability and diminished perceived control. Consequently, resource depletion triggered by incivility is magnified, increasing the probability of retaliatory or withdrawal-based destructive behaviors.

Second, acquiescent silence and defensive silence mediate the effects of digital incivility on CWB. In digital settings, acquiescent silence often arises when employees repeatedly encounter ignored messages, opaque platform rules, and AI-driven rejection, thereby producing learned helplessness. In contrast, defensive silence arises from traceable communication records, heightened digital transparency, and fear of negative algorithmic evaluations. These two silent responses reflect distinct motivational pathway, passive resignation versus strategic avoidance, and jointly explain why digital incivility escalates CWB.

Third, creativity shows the opposite moderating effects in digital contexts. For acquiescent silence, highly creative employees experience stronger cognitive–emotional conflict when suppressed, especially in environments with algorithmic constraints or limited expressive channels, thereby intensifying CWB. For defensive silence, creativity helps employees reinterpret digital stressors, develop alternative strategies, and buffer their destructive tendencies. This duality demonstrates that creativity, within digital work ecosystems, operates as a “context-dependent cognitive resource” rather than a universally positive asset.

## Implications

### Theoretical implications

Based on the framework of workplace incivility, this study systematically integrates the typological mechanisms of silence with the dynamic pathways of employee creativity, constructing a cognition–motivation differentiated model that explains the formation of CWB in resource-threatening contexts. Grounded in COR theory, this study responds directly to the existing theoretical neglect of psychological response patterns in digitally intensified workplace stressors. It offers multilayered extensions to the understanding of negative workplace behaviors.

First, we reconceptualized the consequential logic of workplace incivility theory by positioning incivility, as both a trigger of emotional resource depletion and an instigator of systemic cognition–motivation collapse. Previous studies have predominantly emphasized burnout or attitudinal withdrawal as outcomes of incivility (Liu et al., 2019; Moon and Morais, 2022), but have insufficiently examined how incivility initiates deeper behavioral coping mechanisms that culminate in destructive outcomes. This study identifies silence not as a passive byproduct but as an active psychological stress pathway arising from failed resource regulation by incorporating acquiescent and defensive silence as mediators. This insight extends the theoretical domain of incivility’s consequences beyond mere emotional impairment to the systemic “disintegration of trust and communication mechanisms,” which is particularly salient in digitalized work settings.

Second, this study empirically distinguishes functional heterogeneity within silence behavior theory and advances the contextual application of silence typologies in digital workplaces. Although silence has been classified into acquiescent, defensive, and other forms, most empirical studies continue to treat silence as a homogeneous construct (Chen and Li, 2026; Rodriguez and Zhou, 2023). These findings demonstrate that acquiescent silence and defensive silence stem from fundamentally distinct psychological mechanisms—with the former rooted in resignation and learned helplessness, thus provoking retaliatory, destructive tendencies. The latter reflects strategic self-protection, leading to tactical disengagement. This differentiation propels studies on silence beyond “unified modeling” toward a “conditional, context-sensitive behavioral regulation system,” which is particularly relevant in environments characterized by digital monitoring, algorithmic evaluation, and high information transparency.

Third, this study challenges the traditional assumption that creativity is a universally positive and stable resource (Chang and Chen, 2020), revealing its paradoxical effects on behavioral regulation under different silence motivations. In AS contexts, creativity intensifies CWB by amplifying cognitive–emotional dissonance and facilitating “destructive imagination.” In contrast, in defensive silence contexts, creativity functions as cognitive capital, enabling emotional reframing and psychological restoration and mitigating the likelihood of destructive behaviors. This paradox highlights that creativity, although cognitively advantageous, is constrained by emotional drivers (Khalil et al., 2019). Thus, our findings contribute to emerging discussions on the “double-edged nature of positive psychological resources” and call for a reevaluation of creativity management in digital and resource-strained environments.

Finally, this study extends the boundaries of COR theory within complex psycho-logical–behavioral chains by constructing and validating an integrated moderated-mediation model: “silence typology × creativity moderation × CWB outcomes.” The proposed multistage process, “threat source → behavioral response → creativity regulation → behavioral outcome,” offers a replicable and theoretically extensible frame-work for future studies. Additionally, it provides empirical support for the COR theory’s proposition that resource loss shapes emotional states and dynamically drives behavioral evolution, particularly in technology-mediated and digitally intensified stress environments.

### Practical implications

Beyond its theoretical contributions, this study provides systematic and forward-looking implications for organizational management, particularly in increasingly digitalized work environments. The findings offer an integrated and actionable framework for behavioral governance in modern organizations by uncovering how workplace incivility, including its emerging digital forms, shapes employee CWB through differentiated mechanisms of silence and by illustrating the double-edged moderating role of creativity.

The results illustrate that even seemingly “low-intensity” forms of incivility, such as neglect, sarcasm, delayed digital responses, ambiguous online messages, and algorithm-induced unfairness, can gradually erode employees’ psychological safety and trust in the workplace, ultimately triggering broader negative behavioral patterns through the silence–CWB pathway. This indicates that workplace incivility should no longer be regarded as a minor interpersonal conflict or stylistic inconsistency, but rather as a subtle but consequential governance risk, especially in digitally mediated communication environments where incivility can spread more rapidly and invisibly. Accordingly, managers should embed behavioral civility into their organizational culture through a combination of formal norms and informal technology-enabled feedback mechanisms. Establishing explicit behavioral boundaries, incorporating civility-related indicators into employee experience surveys, and monitoring perceived digital incivility can help organizations detect and address early behavioral warning signs.

The study further suggests that both acquiescent silence and defensive silence reflect psychological isolation responses that emerge after exposure to incivility and that such silence is indicative of weakened communication channels, damaged psychological contracts, and resource depletion. Thus, organizations should move beyond the superficial encouragement of voice and develop systematic structures that identify and address silence as a precursor to functional breakdown. Mechanisms, such as anonymous digital feedback platforms, psychological safety tracking, structured cross-level listening sessions, and open leadership behaviors, can collectively nurture a climate in which employees feel motivated to speak up, even in technology-enabled work settings.

Additionally, the findings highlight that creativity does not invariably serve as a positive resource. In psychologically constrained environments, especially those shaped by unaddressed incivility, creativity may amplify rather than mitigate deviant behavior. In acquiescent silence contexts, creativity can intensify cognitive–emotional conflict and heighten CWB tendencies. In contrast, in defensive silence contexts, creativity operates as cognitive capital, helping employees regulate emotions and reinterpret adverse situations, thereby weakening the link between silence and CWB. These results underscore the need to align innovation expectations with employees’ psychological conditions. Organizations should integrate psychological monitoring into innovation management, create safe spaces for creative expression, and incorporate emotional regulation and cognitive reframing into talent development to prevent creativity from evolving into a destructive imagination.

Finally, the moderated mediation model validated in this study demonstrated that CWB arises from the interaction of environmental factors (organizational and digital incivility), psychological responses (silence), and individual cognitive resources (creativity). Therefore, organizations that rely on fragmented or short-term interventions are unlikely to achieve meaningful improvements. Instead, a multi-layered, psychologically embedded management architecture is essential. This includes minimizing incivility through cultural audits and leadership assessments; optimizing voice systems and communication processes to strengthen emotional–informative feedback loops; and dynamically assessing employees’ psychological states and resource mobilization, particularly among highly creative individuals, to ensure that cognitive resources are directed toward constructive rather than deviant pathways. Such a comprehensive approach enables organizations to transition from reactive intervention to structural prevention, fostering governance that is more precise, human-centered, and aligned with long-term organizational resilience and sustainability in digital ecosystems.

## Limitations and future research

This study employed a cross-sectional research design to collect data on all the variables at a single point in time. Although this approach allows for the identification of significant associations among workplace incivility, silence, and CWB, it limits the ability to draw causal inferences. In digitalized work environments, these variables may interact dynamically; for example, employees’ CWB may reinforce digital incivility (e.g., delayed replies, passive resistance, or refusal to collaborate through digital platforms). Therefore, future studies should adopt longitudinal, multi-wave, or digital-behavior-log designs to clarify the temporal order and strengthen the causal explanations.

Methodologically, this study relied primarily on self-reported data. Despite the use of anonymity, variable masking, and neutral wording, self-reporting bias can be more pronounced in digital contexts. Employees may under-report their CWB due to perceived digital surveillance or over-report incivility due to emotionally amplified platform interactions. Future studies should incorporate multi-source data, such as digital interaction logs, supervisor and peer ratings, cross-validated behavioral assessments, and objective system record, to enhance measurement objectivity and reliability.

Our sample consisted of employees from digitalized workplaces in Guangdong, Sichuan, and Shandong, spanning IT services, digital manufacturing, and platform-based firms. Although this approach provides valuable insights, its representativeness remains limited. Variations in digital maturity, regional cultures, technological intensity, and platform governance rules may influence how employees experience digital incivility and respond to it behaviorally. Industry-specific features, such as algorithmic management in the tech sector or digital workflow rigidity in manufacturing, may also shape silence and CWB patterns. Future studies should expand sampling across regions, industries, and organizations at varying levels of digitalization to improve external validity.

Additionally, while this study focused on acquiescent silence and defensive silence as mediators and examined creativity as a contextual resource in digital environments, these constructs did not exhaust all mechanisms through which digital-era incivility may influence CWB. Other factors, such as digital fatigue, technostress, platform fairness, algorithmic transparency, psychological safety, and organizational identification, may also play meaningful roles. Future studies should incorporate these psychological and structural variables to develop a comprehensive, nuanced “digital resource depletion–regulation–behavior” model that deepens our understanding of behavioral evolution within digital work ecosystems.

## Data Availability

The original contributions presented in the study are included in the article/supplementary material, further inquiries can be directed to the corresponding authors.
